# The prevalence of and perception toward mental illness: a cross-sectional study among Indonesian mental health nurses

**DOI:** 10.1186/s12888-023-05063-6

**Published:** 2023-08-07

**Authors:** Marthoenis Marthoenis, Hasmila Sari, Martina Martina, Rudi Alfiandi, Rini Asnurianti, Hasniah Hasniah, Siti Dara Safitri, Liza Fathiariani

**Affiliations:** 1 Department of Psychiatry and Mental Health Nursing, Universitas Syiah Kuala, Banda Aceh, 23111 Indonesia; 2 Poltekkes Aceh, Banda Aceh, Indonesia; 3 Aceh Provincial Health Office, Banda Aceh, Indonesia; 4 Aceh Psychiatric Hospital, Banda Aceh, Indonesia

**Keywords:** Anxiety, Depression, Perception, Attitude, Mental illness

## Abstract

**Background:**

The emergence of the Coronavirus disease 2019 (Covid-19) pandemic has affected nurses’ mental and psychological health. This study investigates the prevalence of depression, anxiety, and stress among Indonesian mental health nurses and their perception of mental illness.

**Methods:**

A cross-sectional study was conducted shortly before the height of the Covid-19 outbreak in Indonesia. The data were collected using the 21 items of the Depression, Anxiety, and Stress Scale (DASS-21), the questionnaire on perception toward mental illness, and demographic information.

**Results:**

Approximately 2.5%, 6.5%, and 1.9% of the nurses had the symptoms of depression, anxiety, and stress, respectively. The vast majority of them perceive that society should treat well people with mental illness (94.8%) and that the government should protect them (94.8%). More than half also believe that they can eat anything but seldom get physically ill (62.1%) and that in Islam, people with mental illness are innocent and are destined for paradise (61.1%).

**Conclusions:**

A considerably low prevalence of mental distress was discovered, which might be attributed to the nurses’ implementation of mental health skills and effective coping mechanisms. Further training and awareness-raising campaigns are needed to address their misconceptions about mental illness.

## Introduction

The Covid-19 pandemic has impaired health services globally and prevented healthcare providers from interacting face-to-face with patients. In addition, it deteriorates not only the mental health condition of the patients but also of healthcare providers [1, 2], especially the nurses [3, 4]. A Systematic Review and Meta-analysis of 93 studies published between January and September 2020 discovered a pooled prevalence rate of 35%, 37%, and 43% of depression, anxiety, and stress among nurses, respectively [5]. Meanwhile, according to a study conducted in Indonesia, moderate to extremely severe depression, anxiety, and stress prevalence was 8.5%, 20.6%, and 6.3%, respectively [4]. Therefore, the burden of mental distress among Indonesian nurses is relatively lower than reported in the pooled rate of a meta-analysis [5].

Furthermore, the presence of mental symptoms was a factor associated with healthcare workers’ work performance and mental health. The symptoms include depression, anxiety, occupational stress, and fatigue [6]. Understanding the nurse’s perception of the illness and the People with mental illness (PWMI) is significant in improving the healthcare service for PWMI.

In Indonesia, mental health nurses (MHN) work in community health centres (PHC), district general hospitals, or health offices. The nurses in PHC attend to the patients directly, either at the PHC or at the patient’s homes, and are also responsible for school and village mental health programs. Furthermore, nurses in the hospital deal with the in-and out-patients, while the other district health officials are responsible for organizing and supervising the works of MHN at the PHC. Despite being called a nurse, some have an educational background in midwifery, psychology, and public health. However, a minimum of two weeks of basic-level community mental health nursing training is required to work as an MHN. Certified MHN provides training from the Indonesian mental health nursing association and the provincial health office.

The nurse’s knowledge, perception, and stigma toward mental illness could influence their attitude and practice in caring for the PWMI. An earlier study in Jordan suggests that more than half of MHN perceived negative attitudes toward the PWMI, considering them dangerous, harmful, immature, and dirty [7]. The Indian nurses also hold a stigmatizing and negative attitude toward mental illness, despite their adequate knowledge [8]. The nurses are also less likely to deal with mental illness problems in a primary healthcare facility when their knowledge is poor and had a negative attitude toward mental illness [9]. Stigma, negative attitudes, discrimination, fear, lack of skill and knowledge, and pessimistic attitude toward mental treatment and outcomes among MHN could harm the patient’s nursing care [10]. Therefore, acknowledging their knowledge and attitude could be the basis for improving overall mental healthcare, especially during an emergency such as a disease pandemic.

Furthermore, previous studies found that healthcare service providers suffered numerous mental problems while caring for Covid-19 patients [5, 11]. However, only a few reports are available on the mental health condition of nurses attending to patients with mental illness [12, 13] and their perception of it. Therefore, this study aims to estimate the prevalence rate of depression, anxiety, and stress among Indonesian mental health nurses and to examine their awareness, particularly their capacity to acknowledge psychological factors as causes of mental illness, even within a context where mental health issues are predominantly attributed to religious factors.

## Methods

### Study design and participants

A cross-sectional study was conducted in the fourth week of May 2021 among nurses working in mental healthcare programs in Aceh province, Indonesia. Out of the 365 nurses invited, as many as 323 responded and filled in the demographic and DASS-21 questionnaires (response rate = 88.5%), and 211 filled out the perception questionnaire (response rate = 57.8%). The study’s inclusion criteria required participants to meet the following conditions: being officially registered as a mental health nurse working in the community, maintaining their position during the study, and attending the official training on community mental health nursing. The director of the mental health program at the district health office in Aceh province invited the respondents.

### Data collection and measures

A Google form was designed to collect information on the respondents’ age, gender, education, marital status, work settings, perception, experience of interaction with the patients during the Covid-19 pandemic, and their history of Covid-19 vaccination. The Google Form was shared with the participants’ WhatsApp numbers and the MHN WhatsApp group in each district. The presence of depression, anxiety, and stress was assessed using the Indonesian version of the Depression Anxiety and Stress Scale (DASS-21) [14], which has previously been used among Indonesian nurses [4]. The scale has 21 items, with seven questions for each depression, anxiety, and stress. Furthermore, each question has four possible answer options, scored 0 = did not apply to me at all, 3 = applied to me very much, or most of the time. The score of each answer was multiplied by two to comply with the original DASS-42 scoring system. For depression, scores of 14–20, 21–27, and 28 or greater were considered “moderate,“ “severe,“ and “extremely severe,“ respectively. Meanwhile, for the anxiety scale, scores of 10–14, 15–19, and 20 or greater were considered “moderate,“ “severe,“ and “extremely severe,“ respectively. For the stress scale, the score of 19–25, 26–33, and 34 or greater were considered “moderate,“ “severe,“ and “extremely severe,“ respectively [14]. The DASS-21 scale is a user-friendly screening tool used in various populations and settings, with high reliability [15–17]. In this study, the overall reliability coefficient (Cronbach alpha) of the DASS-21 scale was 0.95, while for each sub-scale was 0.88 for depression and stress and 0.87 for anxiety.

The nurse’s perception regarding mental illness was accessed using a questionnaire developed based on a qualitative study of the same settings [18] and used in a community-based survey [19]. The questionnaire consists of 20 statements about perceptions, attitudes, and beliefs about mental illness. According to Islam and the public-government concern on mental illness, the statements include the cause, behaviour, treatment-seeking preference, and view of mental illness. Each question has five possible answers: completely agree, agree, neutral, disagree, and completely disagree. The answer “completely agree and agree” were combined into “agree,“ and the answers “completely disagree” and “disagree” were combined into “disagree,“ while the “neutral” option remained “neutral.“ The overall reliability coefficient (Cronbach alpha) of the perception questionnaire was 0.89, which indicated a high level of internal consistency or reliability among the questions in the measurement instrument.

### Statistical analysis

The respondents’ demographic and clinical characteristics and perceptions were reported in frequency and percentage. The association between depression, anxiety, stress, and other demographic variables was tested using chi-square. Given that no significant association was found (p < 0.05), the results of the tests were not reported.

## Results

Among the 323 mental health nurses that participated in this study, the majority were female (75%), married (85.8%), had a diploma or bachelor’s in nursing (71.8%), and had been vaccinated against Covid-19 (91.6%). Furthermore, more than half were aged between 22 and 40 years old (61.3%), working in community settings (56%), and had previously participated in community mental health nursing training (54.5%). During the Covid-19 pandemic, nearly half of the participants (47.4%) stated that the frequency of interaction between patients with mental illness and the nurses decreased. In comparison, 41.5% stated that the interaction remains similar to before the pandemic. In addition, the study participants’ prevalence of depression, anxiety, and stress was 2.5%, 6.5%, and 1.9%, respectively. Also, no association between these mental illnesses and other demographic variables was observed. Detail of the demographic and clinical characteristics of the study respondents is presented in Table 1.

**Table 1:** Demographic and clinical characteristics of study respondents

No	Characteristics	Total n (%)
1	Gender	
	Male	81 (25)
	Female	242 (75)
2	Age group	
	22–40	198 (61.3)
	41–58	125 (38.7)
3	Highest Education	
	Diploma or Bachelor in Nursing	232 (71.8)
	Other than Nursing	91 (28.2)
4	Marital Status	
	Unmarried	46 (14.2)
	Married	277 (85.8)
5	Training on mental health attended	
	Never attend	147 (45.5)
	Basic	109 (33.8)
	Intermediate	44 (13.6)
	Advanced	23 (7.1)
6	Work settings	
	Community	181 (56)
	Hospital or health office	142 (44)
7	Depression	
	Yes	8 (2.5)
	No	315 (97.5)
8	Anxiety	
	Yes	21 (6.5)
	No	302 (93.5)
9	Stress	
	Yes	6 (1.9)
	No	317 (98.1)
10	Has been vaccinated (Covid-19)	
	Yes	296 (91.6)
	No	147 (45.5)
11	Frequency of nurse -patient interaction during Covid-19 pandemic?	
	Decreased	153 (47.4)
	Remain the same	134 (41.5)
	Increased	20 (6.2)
	Not Sure	16 (4.9)

Overall, Indonesian MHNs have mixed perceptions and expectations regarding mental illness and the people suffering from it. The vast majority agreed that society should treat the PWMI well and that the government should pay more attention and protection to PWMI (94.8%). Meanwhile, more than half perceive that everyone has a chance to suffer from mental illness (72.5%), that the PWMI’s behaviour is unpredictable (68.3%), that the PWMI can generally recover (62.4%), and that it is difficult to talk to the PWMI (51.2%). Less than half perceived that the family of a PWMI is usually looked down upon by the others (46%), and the PWMI often commit violence, such as physically hitting the others (45%). The view regarding the causes of mental illness is also mixed between general and Islamic-related beliefs. For instance, more than half believe that mental illness is caused by a strong desire toward something but not being able to achieve it (66.8%), that in Islam, the PWMIs are innocent and destinated for paradise (61.1%), and that mental illness is a trial from God (60.2%).

**Fig. 1:**
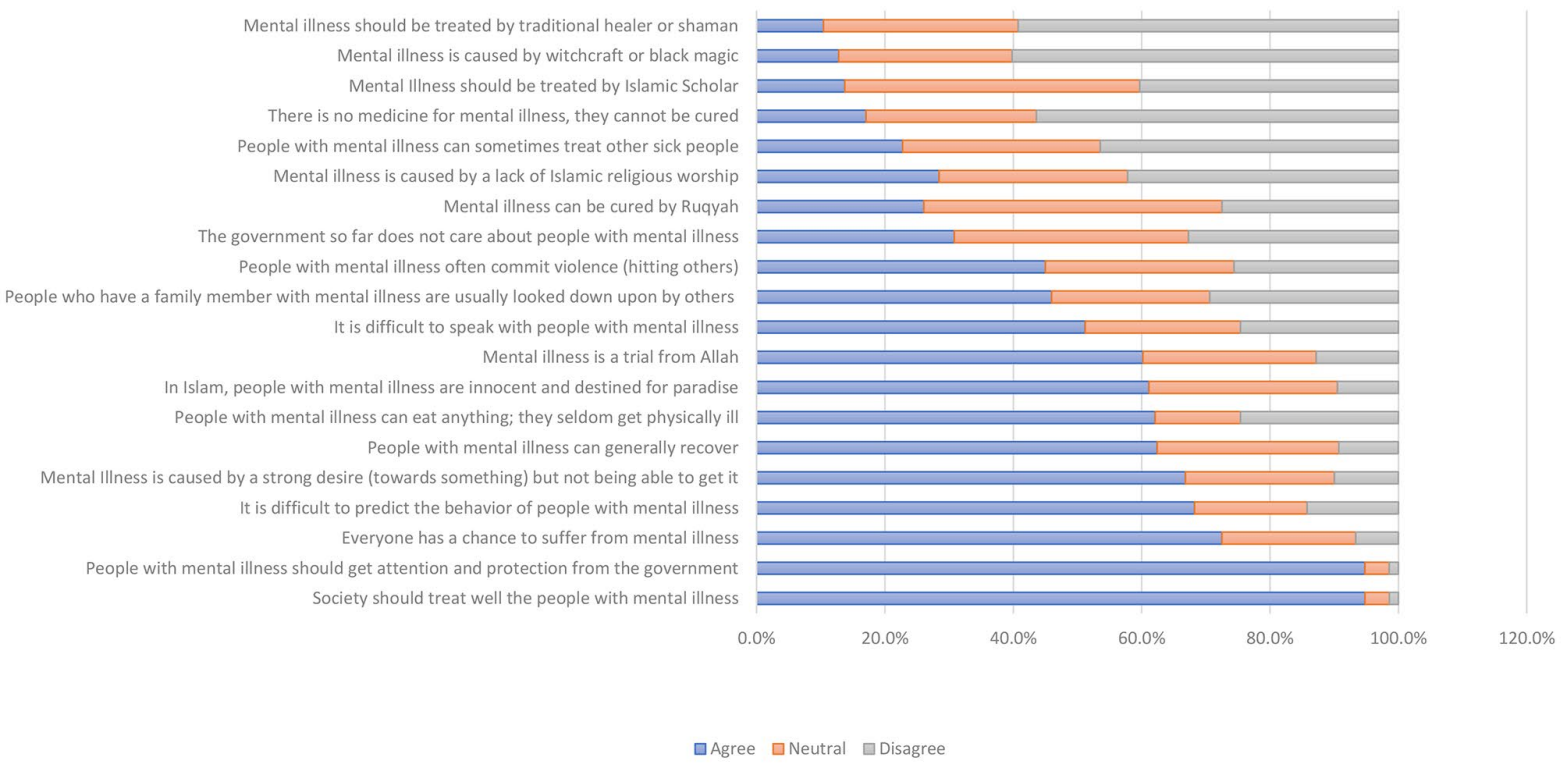
The respondents perception of mental illnes. The questionnaire consists of 20 statements related to perception, attitude, and beliefs about mental illness

Meanwhile, some believe that mental illness is caused by a lack of Islamic religious worship (28.4%) or witchcraft - black magic (12.8%). The presence of non-medical causes beliefs about mental illness engenders the respondents to opt for traditional or cultural mental treatment. Approximately a quarter of them believe that mental illness can be cured by *Ruqyah* (26.1%). In contrast, others suggest the PWMI should be treated by an Islamic scholar (13.7%) or a traditional healer or shaman (10.4%). The worst, some believe that there is no treatment for mental illness (17.1%) or even that the PWMI can sometimes treat other sick people (22.8%). The detail of the answers regarding the respondent’s perception of mental illness and the PWMMI is presented in Fig. 1.

## Discussion

This study found that the prevalence of depression, anxiety, and stress among Indonesian MHN was 2.5%, 6.5%, and 1.9%, respectively. These rates are significantly lower compared to reports from various countries in the form of a systematic review and meta-analysis [5], the rates among nurses working in a Covid-19 referral hospital in Indonesia [4], and the rates among healthcare providers in sub-Saharan African countries [20]. Furthermore, the results found in this study are in line with a report from Portugal, where MHN had fewer symptoms of depression, anxiety, and stress compared to non-MHN [12]. The reduced prevalence of mental distress among MHN could be attributed to their strong comprehension of mental health and preventive measures and their adept utilization of mental health skills to manage distress effectively. An earlier study also suggests that MHN use more mental health promotion strategies than non-MHN [12].

Moreover, throughout the COVID-19 pandemic, healthcare workers in Indonesia received significant support from the government. They were provided COVID-19-related training, personal protective equipment, and special accommodations for those directly handling COVID-19 patients. The government also adjusted their working hours and offered psychological support through counselling. Additionally, healthcare workers were given priority access to vaccination and financial assistance. These comprehensive supports were implemented to alleviate the mental health burden faced by healthcare workers during this challenging time.

Almost half of the respondents also stated that the frequency of interaction with the patients decreased during the pandemic. A reduction in the frequency of interactions indicates the altered intensity of mental health services provided to patients, revealing the tangible impact of the Covid pandemic on the delivery of mental health care. The deterioration of mental health services has also been reported in other countries [21, 22], which forced the MHN to adapt to a new working approach and increase its workload [13]. Digital mental health methods to improve the access and quality of mental health services have been initiated in some studies [23, 24]. However, they are yet to be initiated by community MHN in this study setting.

A significantly high vaccination rate was found among the CMH in this study. Participating in the Covid-19 vaccination program is mandatory for Indonesian healthcare workers. Unfortunately, some MHNs have been recruited temporarily as Covid-19 vaccinators, leaving the primary responsibility of the MHN untouched. A high acceptance rate for vaccination among Indonesian healthcare workers has been predicted in a previous study [25].

Healthcare providers perceive the PWMI as aggressive and unpredictable [26, 27]. The report has been consistent with the finding of our study, where many nurses perceive the PWMI’s behaviour as unpredictable and often commit violence by hitting others. Aggressive and destructive behaviour is prevalent among PWMI in Indonesia, and it has become the reason for instituting *pasung* [28–30]. The CMH should improve the skill of caring for the PWMI, especially those with aggressive behaviour.

The finding of more than half of nurses who perceive that the PWMI could eat anything, such as the leftovers or anything they could find on the street while wandering, and it barely causes them to suffer from a physical problem such as stomachache, seems to be a common belief in this population. The nurses and the general majority population in this setting also have a similar perception [19]. Moreover, the common perception of nurses that it is difficult to predict the behaviour of the PWMI call for further training and educational program for them. The training or education session should not merely be focused on how to treat PWMI but also on common misunderstanding issues regarding mental health and people suffering from it.

Their Islamic religious background influences the nurse’s perception of mental illness and the PWMI. For instance, only 10% of our study respondents did not agree that the PWMI illness is innocent in Islam and that they are destined for paradise. The innocence and destined paradise of the PWMI are common beliefs in this population, which seem to be barely reported in other settings. This belief should affect the nursing process because their religious belief and spiritual coping mediate the relationship between work stress and emotional status, indirectly affecting their service delivery [31]. Furthermore, beliefs that a lack of religious worship causes mental illness and that Islamic scholars could be the treatment option authenticate their views on mental illness. The faith that mental illness is a test from God, the role of supernatural power in causing mental illness, and the importance of the religious aspect of mental treatment have been consistently reported among Muslims [32, 33]. As a mental health professional, the nurse should understand and differentiate the distinctive concept of mental illness from their local – cultural and Islamic belief to the Western understanding of mental illness. After all, promoting a holistic approach has been proposed to improve the recovery of religious patients [33].

In summary, this study revealed a noteworthy decrease in depression, anxiety, and stress rates among Indonesian mental health nurses (MHN). Additionally, it sheds light on mixed perceptions and beliefs about mental illness and individuals with mental illness (PWMI). This particular aspect of the study’s findings is a notable strength, as it addresses an underexplored issue in this context and other Muslim-majority settings. However, the weak association between the study variables warrants additional research, particularly in exploring the potential impact of nurses’ religious-cultural beliefs on enhancing mental health service provision and patients’ recovery. Moreover, this study did not capture certain variables that could influence the mental health of nurses, including socioeconomic status, work environment, and workload management factors. These limitations should be taken into account when conducting future studies.

## Data Availability

Data and materials are available on reasonable request from the corresponding author. To gain access, data requestors must present their analysis plan and sign a data access agreement.
